# 1-(2-Benzoyl-1-phenyl­eth­yl)-4-[(2-hy­droxy-1-naphth­yl)methyl­idene­amino]-3-phenyl-1*H*-1,2,4-triazole-5(4*H*)-thione

**DOI:** 10.1107/S1600536811005721

**Published:** 2011-02-19

**Authors:** Wei Wang, Hong-guo Yao, Yan Gao, Jing-jing Zhang, Xiao-yu Jia

**Affiliations:** a School of Perfume and Aroma Technology, Shanghai Institute of Technology, Shanghai 200235, People’s Republic of China; b School of Chemical Engineering, University of Science and Technology LiaoNing, Anshan 114051, People’s Republic of China

## Abstract

In the title mol­ecule, C_34_H_26_N_4_O_2_S, the hy­droxy group is involved in an intra­molecular O—H⋯N hydrogen bond. The naphthyl ring system and the central triazole ring form a dihedral angle of 37.8 (1)°. The crystal packing exhibits weak inter­molecular C—H⋯O and C—H⋯π inter­actions.

## Related literature

For the pharmacological properties and applications of Mannich bases, see: Joshi *et al.* (2004 ▶); Holla *et al.* (2003 ▶); Negm *et al.* (2005 ▶). For a related structure, see: Wang *et al.* (2011 ▶). For standard bond lengths, see: Allen *et al.* (1987 ▶).
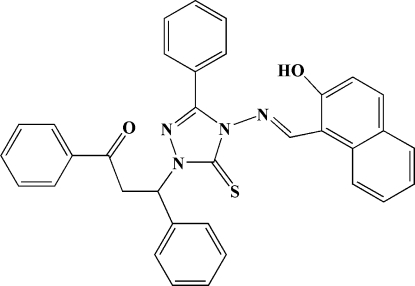


## Experimental

### 

#### Crystal data

C_34_H_26_N_4_O_2_S
*M* *_r_* = 554.65
Monoclinic, 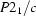

*a* = 13.142 (3) Å
*b* = 21.563 (4) Å
*c* = 10.097 (2) Å
β = 99.22 (3)°
*V* = 2824.4 (10) Å^3^
*Z* = 4
Mo *K*α radiation
μ = 0.15 mm^−1^
*T* = 113 K
0.20 × 0.18 × 0.10 mm

#### Data collection

Rigaku Saturn CCD area-detector diffractometer
Absorption correction: multi-scan (*CrystalClear*; Rigaku/MSC, 2005 ▶) *T*_min_ = 0.970, *T*_max_ = 0.985
23204 measured reflections
4960 independent reflections
4082 reflections with *I* > 2σ(*I*)
*R*_int_ = 0.054

#### Refinement

*R*[*F* ^2^ > 2σ(*F* ^2^)] = 0.045
*wR*(*F* ^2^) = 0.142
*S* = 1.11
4960 reflections
372 parameters
H-atom parameters constrained
Δρ_max_ = 0.34 e Å^−3^
Δρ_min_ = −0.31 e Å^−3^

### 

Data collection: *CrystalClear* (Rigaku/MSC, 2005 ▶); cell refinement: *CrystalClear*; data reduction: *CrystalClear*; program(s) used to solve structure: *SHELXS97* (Sheldrick, 2008 ▶); program(s) used to refine structure: *SHELXL97* (Sheldrick, 2008 ▶); molecular graphics: *SHELXTL* (Sheldrick, 2008 ▶); software used to prepare material for publication: *SHELXTL*.

## Supplementary Material

Crystal structure: contains datablocks global, I. DOI: 10.1107/S1600536811005721/cv5051sup1.cif

Structure factors: contains datablocks I. DOI: 10.1107/S1600536811005721/cv5051Isup2.hkl

Additional supplementary materials:  crystallographic information; 3D view; checkCIF report

## Figures and Tables

**Table 1:** Hydrogen-bond geometry (Å, °) *Cg*1 is the centroid of the C2–C7 ring.

*D*—H⋯*A*	*D*—H	H⋯*A*	*D*⋯*A*	*D*—H⋯*A*
O2—H2⋯N4	0.84	1.85	2.582 (2)	145
C32—H32⋯O1^i^	0.95	2.53	3.196 (2)	127
C14—H14⋯*Cg*1^ii^	0.95	2.60	3.465 (2)	151

Symmetry codes: (i) 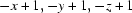; (ii) 
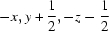
.

**Fig. 1.**
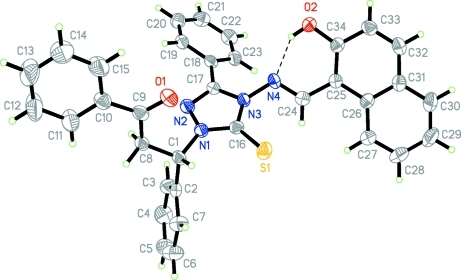
View of (I) showing the atom-labelling scheme. Displacement ellipsoids are drawn at the 60% probability level.

## supplementary crystallographic information

### Comment

We are paying attention to the synthesis and applications of Mannich base due to their comprehensive biological activities (Joshi *et al.*, 2004; Holla *et al.*, 2003; Negm *et al.*, 2005). Recently, we have reported a crystal structure of Mannich base modified by the triazole thione (Wang *et al.*, 2011). Herewith we present the crystal structure of the title compound (I), which is a new Mannich base.

In (I) (Fig. 1), all bond lengths and angles are normal (Allen *et al.*, 1987). An intramolecular O—H···N hydrogen bond (Table 1) results in the formation of a planar (r.m.s. deviation = 0.0094 (2) Å) six-membered ring. This six-membered ring forms a dihedral angle of 36.3 (3)° with the triazole ring. The naphthyl system and central triazole ring form a dihedral angle of 37.8 (1)°.

The weak intermolecular C—H···O and C—H···π interactions (Table 1) contribute to the crystal packing stabilisation.

### Experimental

The title compound was synthesized by the reaction of the chalcone (2.0 mmol) with its corresponding Schiff base, which was inturn obtained by refluxing 4-amino-1-methyl-4*H*-1,2,4-triazole-5-thiol (2.0 mmol), 2-hydroxynaphthalene-1 -carbaldehyde (2.0 mmol) in ethanol. A mixture of Schiff base and chalcone in ethanol was stirring for 24 h.The reaction progress was monitored *via* TLC. The resulting precipitate was filtered off, washed with cold ethanol, dried and purified to give the target product as colorless solid in 84% yield. Crystals of (I) suitable for single-crystal X-ray analysis were grown by slow evaporation of a solution in chloroform-ethanol (1:1).

### Refinement

The hydroxy H atom was located in a differency map, but placed in idealized position with O—H 0.84 Å. C-bound H atoms were positioned geometrically (C—H = 0.95–0.99 Å). All H atoms were refined as riding, with *U*_iso_(H) = 1.2-1.5 *U*_eq_ of the parent atom.

### Crystal data

**Table:**

C_34_H_26_N_4_O_2_S	*F*(000) = 1160
*M**_r_* = 554.65	*D*_x_ = 1.304 Mg m^−^^3^
Monoclinic, *P*2_1_/*c*	Mo *K*α radiation, λ = 0.71073 Å
Hall symbol: -P 2ybc	Cell parameters from 7260 reflections
*a* = 13.142 (3) Å	θ = 1.6–28.0°
*b* = 21.563 (4) Å	µ = 0.15 mm^−^^1^
*c* = 10.097 (2) Å	*T* = 113 K
β = 99.22 (3)°	Prism, colorless
*V* = 2824.4 (10) Å^3^	0.20 × 0.18 × 0.10 mm
*Z* = 4	

### Data collection

**Table:**

Rigaku Saturn CCD area-detector diffractometer	4960 independent reflections
Radiation source: rotating anode	4082 reflections with *I* > 2σ(*I*)
confocal	*R*_int_ = 0.054
Detector resolution: 7.31 pixels mm^-1^	θ_max_ = 25.0°, θ_min_ = 1.6°
φ and ω scans	*h* = −15→15
Absorption correction: multi-scan (*CrystalClear*; Rigaku/MSC, 2005)	*k* = −25→25
*T*_min_ = 0.970, *T*_max_ = 0.985	*l* = −12→11
23204 measured reflections	

### Refinement

**Table:**

Refinement on *F*^2^	Secondary atom site location: difference Fourier map
Least-squares matrix: full	Hydrogen site location: inferred from neighbouring sites
*R*[*F*^2^ > 2σ(*F*^2^)] = 0.045	H-atom parameters constrained
*wR*(*F*^2^) = 0.142	*w* = 1/[σ^2^(*F*_o_^2^) + (0.0813*P*)^2^ + 0.4421*P*] where *P* = (*F*_o_^2^ + 2*F*_c_^2^)/3
*S* = 1.11	(Δ/σ)_max_ = 0.001
4960 reflections	Δρ_max_ = 0.34 e Å^−^^3^
372 parameters	Δρ_min_ = −0.31 e Å^−^^3^
0 restraints	Extinction correction: *SHELXL97* (Sheldrick, 2008), Fc^*^=kFc[1+0.001xFc^2^λ^3^/sin(2θ)]^-1/4^
Primary atom site location: structure-invariant direct methods	Extinction coefficient: 0.0240 (19)

### Special details

**Table:**

Geometry. All e.s.d.'s (except the e.s.d. in the dihedral angle between two l.s. planes) are estimated using the full covariance matrix. The cell e.s.d.'s are taken into account individually in the estimation of e.s.d.'s in distances, angles and torsion angles; correlations between e.s.d.'s in cell parameters are only used when they are defined by crystal symmetry. An approximate (isotropic) treatment of cell e.s.d.'s is used for estimating e.s.d.'s involving l.s. planes.
Refinement. Refinement of *F*^2^ against ALL reflections. The weighted *R*-factor *wR* and goodness of fit *S* are based on *F*^2^, conventional *R*-factors *R* are based on *F*, with *F* set to zero for negative *F*^2^. The threshold expression of *F*^2^ > σ(*F*^2^) is used only for calculating *R*-factors(gt) *etc*. and is not relevant to the choice of reflections for refinement. *R*-factors based on *F*^2^ are statistically about twice as large as those based on *F*, and *R*- factors based on ALL data will be even larger.

### Fractional atomic coordinates and isotropic or equivalent isotropic displacement parameters (Å^2^)

**Table:**

	*x*	*y*	*z*	*U*_iso_*/*U*_eq_	
S1	0.16625 (4)	0.42969 (3)	0.53137 (5)	0.02488 (19)	
O1	0.08098 (12)	0.59528 (7)	0.32929 (16)	0.0304 (4)	
O2	0.56113 (11)	0.45741 (7)	0.40769 (15)	0.0256 (4)	
H2	0.4993	0.4598	0.3708	0.038*	
N1	0.10577 (13)	0.46429 (8)	0.27235 (17)	0.0199 (4)	
N2	0.14223 (13)	0.47373 (8)	0.15336 (17)	0.0218 (4)	
N3	0.26474 (13)	0.43820 (8)	0.30946 (16)	0.0185 (4)	
N4	0.36713 (13)	0.43073 (8)	0.37079 (17)	0.0195 (4)	
C1	−0.00280 (15)	0.47524 (10)	0.2847 (2)	0.0201 (5)	
H1	−0.0055	0.4847	0.3810	0.024*	
C2	−0.06940 (16)	0.41850 (9)	0.2465 (2)	0.0217 (5)	
C3	−0.06416 (17)	0.38507 (11)	0.1301 (2)	0.0285 (5)	
H3	−0.0143	0.3957	0.0754	0.034*	
C4	−0.13163 (19)	0.33608 (11)	0.0934 (2)	0.0339 (6)	
H4	−0.1275	0.3132	0.0140	0.041*	
C5	−0.20463 (18)	0.32055 (11)	0.1720 (2)	0.0336 (6)	
H5	−0.2514	0.2875	0.1458	0.040*	
C6	−0.20963 (19)	0.35305 (11)	0.2889 (3)	0.0351 (6)	
H6	−0.2596	0.3423	0.3433	0.042*	
C7	−0.14105 (17)	0.40157 (10)	0.3265 (2)	0.0279 (5)	
H7	−0.1435	0.4233	0.4078	0.034*	
C8	−0.04660 (15)	0.53104 (9)	0.2024 (2)	0.0200 (5)	
H8A	−0.0383	0.5241	0.1078	0.024*	
H8B	−0.1214	0.5336	0.2053	0.024*	
C9	0.00226 (16)	0.59267 (10)	0.2479 (2)	0.0220 (5)	
C10	−0.04771 (17)	0.65007 (10)	0.1847 (2)	0.0258 (5)	
C11	−0.14383 (19)	0.64937 (11)	0.1051 (3)	0.0436 (7)	
H11	−0.1822	0.6119	0.0939	0.052*	
C12	−0.1845 (2)	0.70293 (13)	0.0417 (4)	0.0623 (10)	
H12	−0.2502	0.7019	−0.0133	0.075*	
C13	−0.1304 (2)	0.75742 (12)	0.0580 (4)	0.0585 (9)	
H13	−0.1582	0.7939	0.0135	0.070*	
C14	−0.0359 (2)	0.75920 (12)	0.1386 (3)	0.0514 (8)	
H14	0.0014	0.7970	0.1507	0.062*	
C15	0.00491 (19)	0.70596 (11)	0.2024 (3)	0.0388 (6)	
H15	0.0699	0.7077	0.2592	0.047*	
C16	0.17801 (16)	0.44309 (9)	0.3722 (2)	0.0191 (5)	
C17	0.24002 (15)	0.45884 (9)	0.1792 (2)	0.0193 (5)	
C18	0.31025 (15)	0.45953 (9)	0.0795 (2)	0.0194 (5)	
C19	0.29602 (16)	0.50478 (10)	−0.0206 (2)	0.0222 (5)	
H19	0.2473	0.5371	−0.0175	0.027*	
C20	0.35328 (17)	0.50241 (11)	−0.1249 (2)	0.0269 (5)	
H20	0.3431	0.5329	−0.1936	0.032*	
C21	0.42476 (17)	0.45594 (10)	−0.1289 (2)	0.0282 (5)	
H21	0.4633	0.4543	−0.2008	0.034*	
C22	0.44066 (18)	0.41152 (10)	−0.0284 (2)	0.0273 (5)	
H22	0.4906	0.3799	−0.0313	0.033*	
C23	0.38430 (17)	0.41304 (10)	0.0757 (2)	0.0234 (5)	
H23	0.3957	0.3827	0.1446	0.028*	
C24	0.38344 (16)	0.39135 (9)	0.4677 (2)	0.0203 (5)	
H24	0.3278	0.3674	0.4899	0.024*	
C25	0.48594 (15)	0.38324 (9)	0.5431 (2)	0.0176 (4)	
C26	0.50280 (16)	0.33939 (9)	0.6524 (2)	0.0207 (5)	
C27	0.42351 (17)	0.30223 (10)	0.6923 (2)	0.0269 (5)	
H27	0.3546	0.3065	0.6473	0.032*	
C28	0.4453 (2)	0.26033 (11)	0.7947 (2)	0.0343 (6)	
H28	0.3911	0.2359	0.8196	0.041*	
C29	0.5456 (2)	0.25274 (11)	0.8635 (3)	0.0362 (6)	
H29	0.5598	0.2230	0.9336	0.043*	
C30	0.62286 (19)	0.28835 (11)	0.8292 (2)	0.0302 (5)	
H30	0.6908	0.2838	0.8772	0.036*	
C31	0.60419 (16)	0.33192 (9)	0.7236 (2)	0.0219 (5)	
C32	0.68548 (17)	0.36873 (11)	0.6870 (2)	0.0263 (5)	
H32	0.7530	0.3644	0.7363	0.032*	
C33	0.66934 (17)	0.40962 (10)	0.5842 (2)	0.0240 (5)	
H33	0.7251	0.4333	0.5615	0.029*	
C34	0.56926 (16)	0.41697 (9)	0.5107 (2)	0.0198 (5)	

### Atomic displacement parameters (Å^2^)

**Table:**

	*U*^11^	*U*^22^	*U*^33^	*U*^12^	*U*^13^	*U*^23^
S1	0.0264 (3)	0.0314 (3)	0.0177 (3)	0.0045 (2)	0.0061 (2)	0.0032 (2)
O1	0.0231 (9)	0.0351 (9)	0.0309 (9)	−0.0044 (7)	−0.0023 (7)	0.0010 (7)
O2	0.0212 (8)	0.0302 (8)	0.0252 (8)	−0.0010 (7)	0.0030 (7)	0.0093 (6)
N1	0.0171 (9)	0.0269 (9)	0.0164 (9)	0.0009 (7)	0.0042 (7)	0.0027 (7)
N2	0.0191 (10)	0.0273 (10)	0.0191 (9)	−0.0007 (7)	0.0034 (7)	0.0012 (7)
N3	0.0155 (9)	0.0240 (9)	0.0161 (9)	0.0012 (7)	0.0025 (7)	0.0015 (7)
N4	0.0157 (10)	0.0243 (9)	0.0179 (9)	0.0026 (7)	0.0006 (7)	−0.0005 (7)
C1	0.0145 (11)	0.0259 (11)	0.0203 (11)	0.0023 (9)	0.0041 (9)	0.0010 (8)
C2	0.0189 (11)	0.0220 (11)	0.0228 (11)	0.0028 (9)	−0.0006 (9)	0.0065 (8)
C3	0.0274 (13)	0.0348 (13)	0.0236 (12)	−0.0045 (10)	0.0050 (10)	0.0019 (10)
C4	0.0367 (15)	0.0356 (13)	0.0284 (13)	−0.0017 (11)	0.0022 (11)	−0.0063 (10)
C5	0.0302 (13)	0.0289 (12)	0.0394 (14)	−0.0082 (10)	−0.0012 (11)	−0.0017 (11)
C6	0.0322 (14)	0.0309 (13)	0.0449 (15)	−0.0064 (10)	0.0143 (12)	−0.0007 (11)
C7	0.0291 (13)	0.0257 (12)	0.0308 (13)	−0.0022 (10)	0.0099 (10)	−0.0018 (9)
C8	0.0153 (11)	0.0251 (11)	0.0203 (11)	0.0016 (9)	0.0047 (9)	0.0014 (8)
C9	0.0175 (12)	0.0288 (12)	0.0205 (11)	−0.0014 (9)	0.0052 (10)	−0.0013 (9)
C10	0.0228 (12)	0.0243 (11)	0.0295 (12)	−0.0005 (9)	0.0019 (10)	−0.0032 (9)
C11	0.0357 (15)	0.0201 (12)	0.0666 (19)	−0.0040 (11)	−0.0176 (13)	0.0025 (12)
C12	0.0496 (18)	0.0281 (14)	0.094 (3)	0.0010 (13)	−0.0344 (17)	0.0071 (15)
C13	0.056 (2)	0.0201 (13)	0.089 (2)	0.0032 (12)	−0.0187 (17)	0.0064 (14)
C14	0.0482 (18)	0.0201 (13)	0.080 (2)	−0.0075 (12)	−0.0076 (16)	−0.0020 (13)
C15	0.0307 (14)	0.0249 (12)	0.0572 (17)	−0.0034 (10)	−0.0032 (12)	−0.0045 (11)
C16	0.0194 (11)	0.0160 (10)	0.0222 (11)	−0.0004 (8)	0.0045 (9)	0.0004 (8)
C17	0.0170 (11)	0.0203 (10)	0.0202 (11)	−0.0019 (8)	0.0018 (9)	0.0014 (8)
C18	0.0152 (11)	0.0236 (11)	0.0190 (11)	−0.0027 (8)	0.0013 (9)	−0.0013 (8)
C19	0.0185 (11)	0.0252 (11)	0.0219 (11)	0.0002 (9)	0.0006 (9)	0.0010 (9)
C20	0.0273 (12)	0.0318 (12)	0.0212 (12)	−0.0027 (10)	0.0026 (10)	0.0023 (9)
C21	0.0289 (13)	0.0355 (13)	0.0217 (12)	−0.0049 (10)	0.0087 (10)	−0.0037 (10)
C22	0.0257 (12)	0.0309 (12)	0.0264 (12)	0.0018 (10)	0.0072 (10)	−0.0049 (9)
C23	0.0228 (12)	0.0244 (11)	0.0219 (12)	0.0010 (9)	0.0002 (9)	0.0007 (9)
C24	0.0206 (12)	0.0198 (10)	0.0209 (11)	0.0002 (8)	0.0047 (9)	−0.0021 (8)
C25	0.0183 (11)	0.0181 (10)	0.0168 (10)	0.0027 (8)	0.0040 (8)	−0.0023 (8)
C26	0.0240 (12)	0.0199 (11)	0.0186 (11)	0.0009 (9)	0.0048 (9)	−0.0002 (8)
C27	0.0260 (13)	0.0278 (12)	0.0268 (12)	−0.0034 (9)	0.0035 (10)	0.0048 (9)
C28	0.0408 (15)	0.0279 (12)	0.0360 (14)	−0.0050 (11)	0.0113 (12)	0.0096 (10)
C29	0.0446 (16)	0.0325 (13)	0.0318 (14)	0.0051 (11)	0.0068 (12)	0.0150 (10)
C30	0.0313 (13)	0.0322 (13)	0.0264 (12)	0.0093 (10)	0.0020 (10)	0.0062 (10)
C31	0.0225 (12)	0.0228 (11)	0.0204 (11)	0.0056 (9)	0.0032 (9)	−0.0012 (8)
C32	0.0200 (12)	0.0322 (12)	0.0252 (12)	0.0034 (9)	−0.0010 (9)	−0.0008 (9)
C33	0.0189 (12)	0.0287 (12)	0.0250 (12)	0.0003 (9)	0.0057 (9)	−0.0007 (9)
C34	0.0235 (12)	0.0210 (10)	0.0153 (10)	−0.0003 (9)	0.0036 (9)	−0.0015 (8)

### Geometric parameters (Å, °)

**Table:**

S1—C16	1.665 (2)	C13—C14	1.372 (4)
O1—C9	1.215 (3)	C13—H13	0.9500
O2—C34	1.349 (2)	C14—C15	1.382 (4)
O2—H2	0.8400	C14—H14	0.9500
N1—C16	1.349 (3)	C15—H15	0.9500
N1—N2	1.378 (2)	C17—C18	1.471 (3)
N1—C1	1.471 (3)	C18—C19	1.396 (3)
N2—C17	1.310 (3)	C18—C23	1.402 (3)
N3—C17	1.377 (3)	C19—C20	1.390 (3)
N3—C16	1.394 (3)	C19—H19	0.9500
N3—N4	1.397 (2)	C20—C21	1.379 (3)
N4—C24	1.287 (3)	C20—H20	0.9500
C1—C2	1.518 (3)	C21—C22	1.387 (3)
C1—C8	1.522 (3)	C21—H21	0.9500
C1—H1	1.0000	C22—C23	1.380 (3)
C2—C7	1.384 (3)	C22—H22	0.9500
C2—C3	1.390 (3)	C23—H23	0.9500
C3—C4	1.391 (3)	C24—C25	1.448 (3)
C3—H3	0.9500	C24—H24	0.9500
C4—C5	1.381 (3)	C25—C34	1.396 (3)
C4—H4	0.9500	C25—C26	1.444 (3)
C5—C6	1.383 (3)	C26—C31	1.418 (3)
C5—H5	0.9500	C26—C27	1.423 (3)
C6—C7	1.393 (3)	C27—C28	1.369 (3)
C6—H6	0.9500	C27—H27	0.9500
C7—H7	0.9500	C28—C29	1.398 (4)
C8—C9	1.515 (3)	C28—H28	0.9500
C8—H8A	0.9900	C29—C30	1.361 (3)
C8—H8B	0.9900	C29—H29	0.9500
C9—C10	1.495 (3)	C30—C31	1.412 (3)
C10—C11	1.384 (3)	C30—H30	0.9500
C10—C15	1.387 (3)	C31—C32	1.426 (3)
C11—C12	1.385 (3)	C32—C33	1.353 (3)
C11—H11	0.9500	C32—H32	0.9500
C12—C13	1.370 (4)	C33—C34	1.411 (3)
C12—H12	0.9500	C33—H33	0.9500
			
C34—O2—H2	109.5	C10—C15—H15	119.6
C16—N1—N2	113.59 (16)	N1—C16—N3	102.42 (16)
C16—N1—C1	124.56 (17)	N1—C16—S1	128.11 (16)
N2—N1—C1	121.84 (17)	N3—C16—S1	129.41 (16)
C17—N2—N1	104.91 (17)	N2—C17—N3	110.08 (18)
C17—N3—C16	108.93 (16)	N2—C17—C18	124.36 (19)
C17—N3—N4	121.57 (16)	N3—C17—C18	125.38 (18)
C16—N3—N4	127.36 (16)	C19—C18—C23	119.5 (2)
C24—N4—N3	116.37 (17)	C19—C18—C17	118.47 (18)
N1—C1—C2	112.14 (16)	C23—C18—C17	121.73 (19)
N1—C1—C8	111.58 (16)	C20—C19—C18	119.8 (2)
C2—C1—C8	110.26 (17)	C20—C19—H19	120.1
N1—C1—H1	107.5	C18—C19—H19	120.1
C2—C1—H1	107.5	C21—C20—C19	120.2 (2)
C8—C1—H1	107.5	C21—C20—H20	119.9
C7—C2—C3	119.2 (2)	C19—C20—H20	119.9
C7—C2—C1	118.89 (19)	C20—C21—C22	120.3 (2)
C3—C2—C1	121.87 (19)	C20—C21—H21	119.9
C2—C3—C4	120.2 (2)	C22—C21—H21	119.9
C2—C3—H3	119.9	C23—C22—C21	120.3 (2)
C4—C3—H3	119.9	C23—C22—H22	119.8
C5—C4—C3	120.2 (2)	C21—C22—H22	119.8
C5—C4—H4	119.9	C22—C23—C18	119.8 (2)
C3—C4—H4	119.9	C22—C23—H23	120.1
C4—C5—C6	120.0 (2)	C18—C23—H23	120.1
C4—C5—H5	120.0	N4—C24—C25	120.45 (19)
C6—C5—H5	120.0	N4—C24—H24	119.8
C5—C6—C7	119.7 (2)	C25—C24—H24	119.8
C5—C6—H6	120.1	C34—C25—C26	119.34 (19)
C7—C6—H6	120.1	C34—C25—C24	120.85 (18)
C2—C7—C6	120.7 (2)	C26—C25—C24	119.80 (18)
C2—C7—H7	119.7	C31—C26—C27	117.64 (19)
C6—C7—H7	119.7	C31—C26—C25	118.60 (19)
C9—C8—C1	115.01 (18)	C27—C26—C25	123.76 (19)
C9—C8—H8A	108.5	C28—C27—C26	120.8 (2)
C1—C8—H8A	108.5	C28—C27—H27	119.6
C9—C8—H8B	108.5	C26—C27—H27	119.6
C1—C8—H8B	108.5	C27—C28—C29	121.3 (2)
H8A—C8—H8B	107.5	C27—C28—H28	119.4
O1—C9—C10	121.2 (2)	C29—C28—H28	119.4
O1—C9—C8	121.3 (2)	C30—C29—C28	119.3 (2)
C10—C9—C8	117.44 (19)	C30—C29—H29	120.3
C11—C10—C15	118.3 (2)	C28—C29—H29	120.3
C11—C10—C9	122.4 (2)	C29—C30—C31	121.4 (2)
C15—C10—C9	119.2 (2)	C29—C30—H30	119.3
C10—C11—C12	120.5 (2)	C31—C30—H30	119.3
C10—C11—H11	119.8	C30—C31—C26	119.6 (2)
C12—C11—H11	119.8	C30—C31—C32	121.3 (2)
C13—C12—C11	120.4 (3)	C26—C31—C32	119.16 (19)
C13—C12—H12	119.8	C33—C32—C31	122.0 (2)
C11—C12—H12	119.8	C33—C32—H32	119.0
C12—C13—C14	119.9 (3)	C31—C32—H32	119.0
C12—C13—H13	120.1	C32—C33—C34	119.7 (2)
C14—C13—H13	120.1	C32—C33—H33	120.2
C13—C14—C15	120.0 (2)	C34—C33—H33	120.2
C13—C14—H14	120.0	O2—C34—C25	123.30 (19)
C15—C14—H14	120.0	O2—C34—C33	115.47 (18)
C14—C15—C10	120.9 (2)	C25—C34—C33	121.22 (19)
C14—C15—H15	119.6		
			
C16—N1—N2—C17	−0.8 (2)	N1—N2—C17—C18	177.69 (18)
C1—N1—N2—C17	−179.26 (17)	C16—N3—C17—N2	−3.0 (2)
C17—N3—N4—C24	−154.97 (19)	N4—N3—C17—N2	−167.49 (16)
C16—N3—N4—C24	43.5 (3)	C16—N3—C17—C18	−178.37 (19)
C16—N1—C1—C2	−90.8 (2)	N4—N3—C17—C18	17.1 (3)
N2—N1—C1—C2	87.5 (2)	N2—C17—C18—C19	34.5 (3)
C16—N1—C1—C8	144.92 (19)	N3—C17—C18—C19	−150.7 (2)
N2—N1—C1—C8	−36.7 (3)	N2—C17—C18—C23	−139.8 (2)
N1—C1—C2—C7	136.1 (2)	N3—C17—C18—C23	35.0 (3)
C8—C1—C2—C7	−98.9 (2)	C23—C18—C19—C20	1.7 (3)
N1—C1—C2—C3	−47.3 (3)	C17—C18—C19—C20	−172.70 (19)
C8—C1—C2—C3	77.7 (2)	C18—C19—C20—C21	−0.7 (3)
C7—C2—C3—C4	1.3 (3)	C19—C20—C21—C22	−0.5 (3)
C1—C2—C3—C4	−175.3 (2)	C20—C21—C22—C23	0.7 (3)
C2—C3—C4—C5	0.4 (4)	C21—C22—C23—C18	0.3 (3)
C3—C4—C5—C6	−1.1 (4)	C19—C18—C23—C22	−1.5 (3)
C4—C5—C6—C7	0.3 (4)	C17—C18—C23—C22	172.7 (2)
C3—C2—C7—C6	−2.2 (3)	N3—N4—C24—C25	−176.06 (16)
C1—C2—C7—C6	174.5 (2)	N4—C24—C25—C34	−1.2 (3)
C5—C6—C7—C2	1.4 (4)	N4—C24—C25—C26	179.30 (18)
N1—C1—C8—C9	−64.8 (2)	C34—C25—C26—C31	−0.2 (3)
C2—C1—C8—C9	169.89 (17)	C24—C25—C26—C31	179.34 (18)
C1—C8—C9—O1	11.4 (3)	C34—C25—C26—C27	−179.18 (19)
C1—C8—C9—C10	−170.98 (18)	C24—C25—C26—C27	0.3 (3)
O1—C9—C10—C11	−171.5 (2)	C31—C26—C27—C28	−0.8 (3)
C8—C9—C10—C11	10.9 (3)	C25—C26—C27—C28	178.3 (2)
O1—C9—C10—C15	10.5 (3)	C26—C27—C28—C29	0.1 (4)
C8—C9—C10—C15	−167.1 (2)	C27—C28—C29—C30	0.9 (4)
C15—C10—C11—C12	2.0 (4)	C28—C29—C30—C31	−1.3 (4)
C9—C10—C11—C12	−175.9 (3)	C29—C30—C31—C26	0.6 (3)
C10—C11—C12—C13	−0.6 (5)	C29—C30—C31—C32	−179.4 (2)
C11—C12—C13—C14	−0.7 (6)	C27—C26—C31—C30	0.4 (3)
C12—C13—C14—C15	0.6 (5)	C25—C26—C31—C30	−178.68 (19)
C13—C14—C15—C10	0.9 (5)	C27—C26—C31—C32	−179.57 (19)
C11—C10—C15—C14	−2.2 (4)	C25—C26—C31—C32	1.4 (3)
C9—C10—C15—C14	175.8 (2)	C30—C31—C32—C33	178.4 (2)
N2—N1—C16—N3	−1.0 (2)	C26—C31—C32—C33	−1.6 (3)
C1—N1—C16—N3	177.48 (17)	C31—C32—C33—C34	0.6 (3)
N2—N1—C16—S1	176.44 (15)	C26—C25—C34—O2	177.88 (18)
C1—N1—C16—S1	−5.1 (3)	C24—C25—C34—O2	−1.6 (3)
C17—N3—C16—N1	2.3 (2)	C26—C25—C34—C33	−0.9 (3)
N4—N3—C16—N1	165.67 (17)	C24—C25—C34—C33	179.63 (18)
C17—N3—C16—S1	−175.07 (16)	C32—C33—C34—O2	−178.16 (19)
N4—N3—C16—S1	−11.7 (3)	C32—C33—C34—C25	0.7 (3)
N1—N2—C17—N3	2.2 (2)		

### Hydrogen-bond geometry (Å, °)

**Table:**

Cg1 is the centroid of the C2–C7 ring.

**Table:**

*D*—H···*A*	*D*—H	H···*A*	*D*···*A*	*D*—H···*A*
O2—H2···N4	0.84	1.85	2.582 (2)	145
C32—H32···O1^i^	0.95	2.53	3.196 (2)	127
C14—H14···Cg1^ii^	0.95	2.60	3.465 (2)	151

Symmetry codes: (i) −*x*+1, −*y*+1, −*z*+1; (ii) −*x*, *y*+1/2, −*z*−1/2.
